# Validation and Reliability of Healthcare Workers’ Knowledge, Attitude, and Practice Instrument for Uncomplicated Malaria by Rasch Measurement Model

**DOI:** 10.3389/fphar.2019.01521

**Published:** 2020-01-10

**Authors:** Nahlah E. Ismail, Nanloh S. Jimam, Maxwell L. P. Dapar, Sohail Ahmad

**Affiliations:** ^1^ Department of Clinical Pharmacy, Faculty of Pharmacy, MAHSA University, Petaling Jaya, Malaysia; ^2^ Department of Clinical Pharmacy and Pharmacy Practice, Faculty of Pharmaceutical Sciences, University of Jos, Plateau State, Nigeria

**Keywords:** Rasch analysis, person-item reliability, fit index, knowledge, attitude and practice, uncomplicated malaria

## Abstract

**Background:** This study assessed the validity and reliability of healthcare workers’ knowledge, attitudes, and practices instrument for uncomplicated malaria (HKAPIUM) for evaluation of healthcare workers’ knowledge, attitudes, and practices (KAP) on uncomplicated malaria management in primary healthcare (PHC) facilities in Plateau state, Nigeria.

**Methods:** Relevant variables from literature, malaria treatment guidelines for Nigeria, and World Health Organization (WHO) were used to generate and present the items for the draft HKAPIUM scale, which was first screened by six experts before administered to 121 respondents who filled and returned immediately. The data were sorted and analyzed using Rasch measurement model (Bond & Fox software^®^).

**Results:** The outcome of the initial screening showed high items content validity indices (I-CVI) (0.83–1.00) and high scale-CVI (S-CVI) {universal agreement (UA) within the experts (S-CVI/UA) (0.67–0.89) and the average CVI [S-CVI/Ave (0.94–0.98)]} for relevance, clarity, simplicity, and comprehensiveness. The Rasch analysis outputs showed good items’ reliability for the three factors (KAP) > 0.9 with high separation index values of > 2.0; however person reliability were poor (< 0.6) which were confirmed by their low separation values. Goodness of fit statistics indicated nine items not fitting the model based on the suggested fit index values of 0.6 to 1.5, and ± 2 for mean square (MNSQ) and standardized Z-score (Zstds) respectively, and 0.3 to 0.7 for “point-measure correlation coefficients” (PTMEA Corr). Deletion of misfit items resulted in the items and persons’ reliabilities falling above the minimum accepted limit of 0.6, with their separation values were all in the range of 1 and 2 which were acceptable. Similarly, fit index values for MNSQ infit and outfit, and Zstd parameters items in the new scale were all within the acceptable range of 0.6 to 1.5, and ±2 respectively, in addition to the positive PTMEA Corr as further confirmation of the items’ fitness to the model.

**Conclusion:** The reduction of 27-items draft HKAPIUM scale to 18 items was successful with good reliability and fitness to the model.

## Introduction

The use of public primary healthcare (PHC) facilities in Nigeria which is the closest source of healthcare to the rural communities has been shown to be poor, and this has been linked to many factors including lack of drugs in the public PHC facilities and bad healthcare practices including diagnostic practices, prescription and dispensing practices rendered by the PHC workers (Onwujekwe et al., 2010; Abdulraheem et al., 2012). Uzochukwu et al. (2002) had also reported high level of irrational drug prescription in the south-eastern Nigeria by healthcare workers in healthcare facilities where drug revolving fund programs were implemented to augment drugs availabilities. Even though the availability of drugs is not enough, the rational use of drugs should be encouraged and strengthen. Most of the inappropriate management practices for malaria and other common ailments in the country have been linked to the knowledge and behavioral characteristics of the healthcare workers (Bello et al., 2013; Nduka et al., 2013; Okoli et al., 2015; Bamiselu et al., 2016). The use of knowledge, attitude, and practice (KAP) instruments have been reported especially in social sciences and public health to assess such behavior-related attributes (Krentel et al., 2006). In order to maintain a standardized approach and ensure quality during assessment of healthcare workers’ KAP on uncomplicated malaria in PHC facilities, there was a need for the development and validation of KAP instrument. This was necessitated by scanty information on validated relevant scales for such study. Construct validity has been described as the extent to which an instrument measures what it intends to measure correctly (Agarwal, 2011), while the reliability is the degree of consistency or dependability with which an instrument measures the attribute it is designed to measure (Müller et al., 2015). Analysis of instruments for validity and reliability are often carried out using test theory approaches including classical test theory (CTT) and modern response theory (MRT), also known as item response theory (IRT) (Thompson, 2009; Müller et al., 2015).

Though both CTT and IRT are important in assessing the fitness of data/items to the measuring instrument, the CTT has the test instrument as its basis while the concept of IRT looks beyond the underlying traits which are producing the test instrument performance (Wirth and Edwards, 2007; Thompson, 2009). It is a model for the design and evaluation of relationships between the latent trait of interest, and the observed variables (items) in addition to determine how the test instrument as a whole relates to the latent trait (Wirth and Edwards, 2007; Thompson, 2009). The use of Rasch method has been considered a better option for such analysis since it takes both persons and items’ attributes into account, and it is also convenient for this kind of studies (Rasch, 1980; Zamalia et al., 2013; Janssen et al., 2014; Akram et al., 2015; Müller et al., 2015). The patterns of individuals’ responses to items (consistent or idiosyncratic) are indicated by person fit index value. On the other hand, item fit index indicates the usefulness of the items in providing continuum that could be considered useful to the respondents. Item may misfit due to its complexity, confusing nature, and in some cases because it may not be the rightful item for measuring such construct. The present study determined the items and person reliability, and the validity of healthcare workers’ KAP instrument for uncomplicated malaria (HKAPIUM) scale using Rasch measurement model.

## Methods

### Item Generation and Presentation

Based on the purpose of the study, the first stage of the instrument development involved generation of variables list that best represented healthcare workers’ KAP on uncomplicated malaria management. Such variables were identified and selected based on literature reviews of related journals, Nigeria and World Health Organization (WHO) malaria treatment guidelines (Federal Ministry of Health (FMOH), 2015; Jimam et al., 2015; WHO, 2016). The variables were used to generate statements for the instruments using two approaches including the Likert scale. Terms such as “strongly agree,” “agree,” “neutral,” “disagree,” and “strongly disagree”; and “very often,” “often,” “sometimes,” “rarely,” and “never” were used to describe how strongly respondent feels about the statements. The fixed-choice option was the second approach which involved framing statements such that respondent has to make a fixed choice answer of “yes,” “no” or not sure (Burns et al., 2008). At the end, self-reported draft healthcare workers’ KAP instrument for uncomplicated malaria (HKAPIUM) containing a total of 27 items was developed.

### Description of the Draft 27-Item Healthcare Workers’ Knowledge, Attitude, and Practice Instrument for Uncomplicated Malaria Instrument

**Section 1** contained 16 items to test healthcare workers’ basic knowledge on the cause and transmission, sign and symptoms, diagnosis, and recommended anti-malarial drugs for management of uncomplicated malaria. Their levels of correct responses to the 16 items were assessed using three options of “no,” “not sure,” or “yes” which were scored as 1 for correct response (yes or no), and as 0 for wrong response (yes, no, not sure).

**Section 2:** This subsection had five items for assessing respondents’ attitudes toward uncomplicated malaria and its management. The magnitude of their attitudes were assessed on 5-point Likert scale with scores ranging from 1 = strongly disagree, 2 = disagree, 3 = neutral, 4 = agree, and 5 = strongly agree.

**Section 3** of the draft HKAPIUM scale consisted of six items presented in 5-point Likert scale format for evaluating healthcare workers’ management practices, and their responses were also scored on 5-point Likert scale as 1 = never, 2 = rarely, 3 = sometimes, 4 = often, and 5 = very often.

### Face and Content Validity

The content validity of the draft instrument (27-item HKAPIUM) was qualitatively and quantitatively determined using experts in the field (Ayre and Scally, 2014). The face validity was used to check the appropriateness of the statements’ constructions for each of the items relating to wordings, structures, orderliness, and scoring formats (Creswell, 2014). Based on their observations and suggestions, appropriate amendments were made accordingly, after which clean copies were returned to the same experts together with short 4-point Likert scale and cover letter explaining the purpose of the study, the need for content validation of the research instruments, and the detail description to evaluate the items. They were requested to independently express their views on the instrument regarding its relevance, clarity, simplicity, and comprehensiveness using the short Likert scale (Devon et al., 2007; Rodrigues et al., 2017).

The quantification of experts’ views regarding the content validity of the scales was carried out through content validity index (CVI) approach (Devon et al., 2007; Rodrigues et al., 2017). An item was considered relevant if the item content validity index (I-CVI) was > 0.79, need revision when value falls between 0.70 and 0.79, and rejected when values was < 0.7 (Devon et al., 2007). Similarly, the scale CVI (S-CVI) was estimated through the universal agreement (UA) within the experts (S-CVI/UA) and the average CVI (S-CVI/Ave) methods (Devon et al., 2007; Zamanzadeh et al., 2015). Values of S-CVI/UA ≥ 0.80 and an S-CVI/Ave ≥ 0.90 were considered excellent content validity (Ayre and Scally, 2014; Zamanzadeh et al., 2015).

### Construct Validity and Reliability Using Rasch Measurement Model

#### Study Population and Sampling Methods

The study population consisted of healthcare workers involved in the management of uncomplicated malaria in public PHC facilities of Plateau state, Nigeria. Considering the limited population of healthcare workers, purposive sampling method (Neuman, 2005) was used to recruit respondents from eight selected PHC facilities to participate in the validation of the 27-item draft HKAPIUM instrument. This method was preferred in order to get as many respondents that were available in the selected PHC facilities for the purpose of achieving good number of responses as possible (Kutner et al., 2005).

#### Sample Size Calculation

Absolute sample size of 121 respondents was estimated for the study. This was considered adequate based on report of Linacre (1994) that sample size of as low as 30 to 50 was adequate to run Rasch analysis. Garson (2008) and Habing (2003) had also reported sample size of between 100 and 150 to be adequate for factor analysis. Such low sample size might yield good outputs, especially if related to the person’s reliability and separation index values, as there might be fewer reported lapses made by the respondents compared to larger populations (Linacre, 1994; Linacre, 2012).

#### Data Collection

A draft healthcare professionals’ self-reported instrument containing a total of 27 items earlier developed from list of variables that best-represented healthcare professionals’ KAP as they relate to uncomplicated malaria management were used for data collection. Prior to distribution of the draft instrument to 121 respondents involved in malaria case management in primary healthcare (PHC) facilities in Plateau state, Nigeria, to fill and returned instantly, permission to conduct the study was granted by the Joint Research Review and Ethics Committee, Research Management Centre (RMC), MAHSA University, Malaysia (Ref. number: RMC/EC01/2016; Dated 25/11/2016). Respondents’ information were then extracted, coded, and entered into Microsoft Excel for Rasch analysis.

#### Data Analysis

The Rasch measurement model which is considered as an extension of the CTT was used to describe reliability and fitness of the data to the model using Bond and Fox software^®^ on the 121 healthcare workers’ data (Rasch, 1980; Bond and Fox, 2015). The characteristics of the measurement were evaluated based on the properties of the administered items and the response patterns of the respondents. In the present study, items and persons’ reliability of the constructs and the adequacy of separation indices were evaluated. Fit statistics of the items to the model were also assessed to provide fit scores that showed whether the items and persons’ behaviors were consistent with the expected ones of the model (unidimensionality), and hence the validity of the instrument using the output values (Bond and Fox, 2015).

In the first instance the Rasch analysis outputs for the data extracted using the draft HKAPIUM (27 items) were displayed and interpreted based on certain recommendations: item and person reliability value > 0.8 was good, while values > 0.6 and < 0.8 were considered fair and acceptable, but values < 0.6 were rejected; while the separation index value of > 1 was considered useful for the instrument, and > 2.0 as good (Linacre, 2012). In the case of validity studies, fit statistics parameters including the mean-square infit and outfit values (MNSQs-infit/outfit), the standardized Z values (ZSTDs-infit/outfit), and the point-measure correlation coefficient (PTMEA Corr) were used for assessment. Based on the suggestions, MNSQ infit and outfit value range of between 0.6 and 1.5 was accepted as good for both item and person fitness, the PTMEA Corr value range of 0.3 to 0.7, and the ZStd values of ± 2.0 were also accepted as a measure of fitness (Linacre, 2012; Bond and Fox, 2015). Although, the outfit MNSQ index values are mostly used as indicator of item misfit to model during Rasch model output interpretation because it is un-weighted (Linacre, 2012), in the present study, both the two index values (infit and outfit MNSQ) were considered together with their corresponding ZStd index values in reducing a large number of items into smaller size that could give more meaning to the HKAPIUM scale as a valid research instrument for public use. In addition, the PTMEA Corr was used to check if the items were moving in the same direction with the factors; with positive values were indications that the items were parallel to the factors (Linacre, 2012). During the interpretation of the Rasch analysis statistic outputs, items with index values of two or more parameters outside the normal range were identified as misfitting and were marked for deletion or reframing, and the analysis re-run again to see whether such removal of misfit items had any influence on the model fitness as predicted.

## Results

### Face and Content Validity

It was ensured that only relevant variables were selected and used in the design of the draft instruments. All observations/contributions made on the drafted instruments by medical experts were appropriately utilized in updating the instrument. The results of the content validity study for the 27 items were interpreted through Lynn’s approach (Lynn, 1986), and all the items had CVI (I-CVI) > 0.80 for relevance, clarity, simplicity, and comprehensiveness (**Table 1**). The average CVIs (S-CVI) for relevance, clarity, simplicity, and comprehensiveness for the scale based on the results of the universal agreement (UA) within the experts (S-CVI/UA) and the average CVI (S-CVI/Ave) approaches were in the ranges of 0.67–0.89 and 0.94–0.98, respectively (**Table 1**).

**Table 1:** Content validity of the 27-item draft healthcare workers’ knowledge, attitudes, and practices instrument for uncomplicated malaria scale (N = 6).

Description	Relevance		Clarity		Simplicity		Comprehensiveness	
	n	I-CVI	n	I-CVI	n	I-CVI	n	I-CVI
Item 1	6	1	6	1	5	0.83	6	1
Item 2	5	0.83	5	0.83	5	0.83	5	0.83
Item 3	6	1	6	1	6	1	6	1
Item 4	6	1	6	1	6	1	6	1
Item 5	6	1	6	1	6	1	6	1
Item 6	6	1	6	1	6	1	6	1
Item 7	6	1	6	1	5	0.83	5	0.83
Item 8	6	1	6	1	6	1	6	1
Item 9	6	1	5	0.83	6	1	6	1
Item 10	6	1	5	0.83	5	0.83	5	0.83
Item 11	5	0.83	5	0.83	5	0.83	5	0.83
Item 12	6	1	6	1	6	1	6	1
Item 13	6	1	6	1	6	1	6	1
Item 14	6	1	6	1	6	1	6	1
Item 15	6	1	6	1	6	1	6	1
Item 16	6	1	5	0.83	5	0.83	6	1
Item 17	6	1	6	1	6	1	6	1
Item 18	5	0.83	5	0.83	5	0.83	5	0.83
Item 19	6	1	6	1	6	1	6	1
Item 20	6	1	6	1	6	1	6	1
Item 21	6	1	5	0.83	6	1	6	1
Item 22	6	1	6	1	6	1	6	1
Item 23	6	1	6	1	5	0.83	5	0.83
Item 24	6	1	6	1	6	1	6	1
Item 25	6	1	6	1	6	1	6	1
Item 26	6	1	6	1	6	1	6	1
Item 27	6	1	6	1	5	0.83	5	0.83
S-CVI/Ave		0.98		0.96		0.94		0.96
Total agreement		24		20		18		20
S-CVI/UA		0.89		0.74		0.67		0.74
Total agreement (%)		88.89		74.07		66.67		74.07

### Reliability and Fit Statistics for Draft Healthcare Workers’ Knowledge, Attitudes, and Practices Instrument for Uncomplicated Malaria (27 Items) Using Rasch Model

The summary of items and persons’ reliability and the separation indices for the draft HKAPIUM scale as generated by Rasch analysis was presented in **Table 2** below. The items’ reliability for the three factors (knowledge, attitude, and practice) were all > 0.9 with high separation index values above the minimum acceptable value of > 2.0, however the person reliability index values were poor (< 0.6), which were also seen in the respective separation index values of the constructs.

**Table 2:** Reliability and separation indices for draft healthcare workers’ knowledge, attitudes, and practices instrument for uncomplicated malaria containing 27 items using Rasch model (N = 121).

Constructs	ID Item	Item measure		Person measure	
		Reliability	Separation	Reliability	Separation
Knowledge	Items 1–16	0.95	4.57	0.49	0.97
Attitude	Items 17–22	0.98	7.40	0.54	1.08
Practice	Items 23–27	0.98	6.54	0.47	0.94

Based on the recommendation of Bond and Fox (Bond and Fox, 2015) for acceptable fit index value ranges, 9 items were identified misfits to the model (items 2, 3, 11, 13, 14, 15, 21, 23, and 24), and were marked for deletion from the instrument, leaving a total of 18 items (**Table 3**).

**Table 3:** Items fit and misfit indices for draft healthcare workers’ knowledge, attitudes, and practices instrument for uncomplicated malaria containing 27 items (N = 121).

Item No.		Infit MNSQ	Outfit MNSQ	Infit Zstd	Outfit Zstd	PTMEA
**Knowledge**						
1	*Plasmodium falciparum* is the most common parasite that causes malaria in Nigeria	1.45	1.16	2.2	0.8	0.24
2	*Plasmodium malariae* is the most common parasite that causes malaria in Nigeria	1.08	1.08	**2.8**	**2.3**	0.24
3	Malaria parasite is transmitted by male *anopheles* mosquitoes	0.47	0.51	**−6.4**	**−5.7**	**−0.03**
4	Malaria parasite is transmitted by female *anopheles* mosquitoes	1.31	1.25	1.1	0.8	0.16
5	Fever is a symptom of uncomplicated malaria	1.33	1.36	1.6	1.5	0.15
6	Body weakness is a symptom of uncomplicated malaria	1.19	1.00	1.2	0.1	0.39
7	Headache is a symptom of uncomplicated malaria	1.07	0.89	0.5	−0.6	0.53
8	Anemia is a symptom of uncomplicated malaria	1.05	0.90	0.4	−0.6	0.50
9	Confusion is a symptom of uncomplicated malaria	1.03	0.90	0.3	−0.7	0.58
10	Increased respiratory rate is a symptom of uncomplicated malaria	1.01	0.92	0.1	−0.7	0.56
11	Convulsion is a symptom of uncomplicated malaria	0.88	**1.86**	−1.2	**−2.3**	0.38
12	Light microscopy is a method of malaria diagnosis in Nigeria	1.32	1.20	2.3	1.3	0.38
13	Rapid diagnostic test (RDTs) is a method of malaria diagnosis in Nigeria	1.37	1.56	**2.5**	**3.1**	0.08
14	Clinical diagnosis is a method of malaria diagnosis	1.07	**2.03**	0.6	**2.3**	0.33
15	Chloroquine is a drug for uncomplicated malaria in Nigeria	0.59	0.59	**−4.6**	**−4.4**	0.35
16	Artemisinin–based combination therapy (ACTs) is a drug for uncomplicated malaria in Nigeria	1.44	0.99	1.8	0.0	0.34
**Attitudes**						
17	Malaria is a serious problem to the society	0.94	1.30	−0.3	1.7	0.37
18	Primary health care clinics can provide good care for malaria illnesses	0.71	0.86	−2.0	−0.9	0.61
19	Appearance of the clinic and personality of the providers influences patients’ patronage to facilities	0.93	0.85	−0.4	−0.8	0.52
20	Being polite to your clients or the care giver is a key ingredient when attending to them in the PHC	1.23	1.19	1.9	1.5	0.63
21	Injections are the most effective means of treatment for malaria	1.04	**2.89**	0.3	**−2.31**	0.49
22	Malaria can be prevented by educating the community on preventive measures	0.81	1.0	−0.8	−0.5	0.52
**Practices**						
23	I wait for laboratory results before administering anti-malarial drugs to patients	1.16	**1.72**	1.4	**3.3**	0.30
24	I always dispensed anti-malarial drugs to patients in accordance to the available money they have	0.90	**1.74**	−0.6	**-2.7**	0.69
25	I always write instruction to patients on how to take anti-malarial drugs	0.86	0.77	−1.0	−1.5	0.63
26	I ensure that patients are counseled on how to use anti-malarial drugs and it expected side effects	0.94	0.74	−0.4	−1.7	0.68
27	I do advise my patients to always sleep under mosquitoes’ nets	1.49	1.35	2.0	2.1	0.27

The bolded figures in Table 3 indicated items whose some of the fit indices were outside recommended range.

### Reliability and Fit Statistics for Healthcare Workers’ Knowledge, Attitudes, and Practices Instrument for Uncomplicated Malaria (18 Items) After Deletion of Misfit Items

After deletion of the misfit items, Rasch analysis was re-run again to see whether such removal of misfit items had any influence on the model reliability and fitness as predicted by the model. The outcome showed the knowledge construct reliability as 0.90, while those for attitude and practice constructs were 0.75 and 0.65, respectively. Values of their corresponding separation indices were 2.99 (knowledge), 1.73 (attitude), and 1.11 (practice). Similarly, the person reliability measures for knowledge, attitude, and practice were 0.64, 0.61, and 0.70 in that order and hence all within the acceptable range of 0.6 to 0.8 values. The person separation index values were in the range of 1 and 2 (**Table 4**).

**Table 4:** Reliability and separation indices for healthcare workers’ knowledge, attitudes, and practices instrument for uncomplicated malaria containing 18 items using Rasch model (N = 121).

Constructs	ID Item	Item measure		Person measure	
		Reliability	Separation	Reliability	Separation
Knowledge	Items 1–10	0.90	2.99	0.64	1.13
Attitude	Items 11–15	0.75	1.73	0.61	1.01
Practice	Items 16–18	0.65	1.11	0.70	1.54

The generated output of the re-run Rash analysis revealed that the mean square (MNSQ) values of all the 18 items of the three construct (KAP) that make up the HKAPIUM scale were within the ranges of 0.75 to 1.44 (MNSQ infit), and 0.58 to 1.48 (MNSQ outfit); while the corresponding standardized mean (Zstd) values were between −1.4 to +1.5 (infit), and −1.4 to +1.0 (outfit), which were all within the accepted ranges of 0.6 to 1.5 (MNSQ) and ± 2.0 (ZStd) (Linacre, 2012; Bond and Fox, 2015). Similarly, the items polarity measured as PTMEA Corr were all positive, with the majority of them indicating good correlation with their respective constructs, although few were outside the recommended range limits of 0.3 and 0.7 (**Table 5**) (Linacre, 2012).

**Table 5:** Items fit and misfit indices for healthcare workers’ knowledge, attitudes, and practicesinstrument for uncomplicated malaria-18 items (N = 121).

Item No.		Infit MNSQ	Outfit MNSQ	Infit Z std	Outfit Z std	PTMEA
**Knowledge**						
1	*Plasmodium falciparum* is the most common parasite that causes malaria in Nigeria	1.39	1.48	1.5	1.0	0.32
2	Malaria parasite is transmitted by female *anopheles* mosquitoes	0.99	1.00	0.1	0.2	0.30
3	Fever is a symptom of uncomplicated malaria	1.44	0.80	0.7	−0.2	0.30
4	Body weakness is a symptom of uncomplicated malaria	0.98	0.97	−0.1	−0.3	0.40
5	Headache is a symptom of uncomplicated malaria	0.80	0.64	−1.3	−0.6	0.48
6	Anemia is a symptom of uncomplicated malaria	0.84	0.58	−1.1	−0.8	0.49
7	Confusion is a symptom of uncomplicated malaria	0.83	0.62	−1.4	−1.0	0.54
8	Increased respiratory rate is a symptom of uncomplicated malaria	0.94	0.78	−0.5	−0.6	0.53
9	Light microscopy is a method of malaria diagnosis in Nigeria	1.11	0.86	0.9	−0.2	0.45
10	Artemisinin–based combination therapy (ACTs) is a drug for uncomplicated malaria in Nigeria	1.26	0.87	1.0	−0.1	0.36
**Attitudes**						
11	Malaria is a serious problem to the society	1.04	0.78	0.3	−1.3	0.67
12	Primary health care clinics can provide good care for malaria illnesses	1.22	1.17	1.1	1.0	0.60
13	Appearance of the clinic and personality of the providers influences patients’ patronage to facilities	0.75	0.88	−1.2	−0.7	0.75
14	Being polite to your clients or the care giver is a key ingredient when attending to them in the PHC	1.00	0.90	0.1	−0.6	0.71
15	Malaria can be prevented by educating the community on preventive measures	1.20	1.17	1.1	1.0	0.62
**Practices**						
16	I always write instruction to patients on how to take anti-malarial drugs	0.86	0.76	−0.8	−1.4	0.84
17	I ensure that patients are counseled on how to use anti-malarial drugs and it expected side effects	1.20	1.01	1.1	0.1	0.84
18	I do advise my patients to always sleep under mosquitoes’ nets	1.04	0.91	0.3	−0.4	0.82

## Discussions

The importance of carefully selecting the variables for drafting the instrument was to ensure true representation of the KAP constructs that were used in presenting the items in simplified ways for easy understanding by the prospective respondents. The acceptability of the content and certification of the draft instrument by the panel of experts as shown by the high I-CVI and S-CVI/UA values (**Table 1**) was an indication that such instrument might be a good one for assessing healthcare workers’ KAP on uncomplicated malaria (Lynn, 1986; DeVon et al., 2007; Burns et al., 2008).

The importance of Rasch measurement model in assessing the validity and reliability of survey instrument has been recognized (Rasch, 1980; Thompson, 2009; Golino et al., 2014; Müller et al., 2015). Based on the suggested index values for interpretation of Rasch model outputs (Linacre, 2012; Bond and Fox, 2015), 9 items were eliminated from the model leaving a total of 18 items. The reduction in a large number of the items resulted in a slightly negative impact on the items’ reliability. Similar observation was made with the separation values, although, all index values were within acceptable limits (Bond and Fox, 2015), which could be a proof that the measures have the ability to distinguish the items into two or more distinct groups, despite the low index values (Kook and Varni, 2008). The low separation index values of the items might partly be influenced by the sample size of the study population, although, it has been reported that small sample could be enough for validity analysis of instrument using Rasch measurement model (Bond and Fox, 2015), which was evident in the item reliability and separation index values for knowledge. On the contrary, studies have also shown that increasing the sample size might result in the increase and better separation index and reliability of a scale (Linacre, 2012; Golino et al., 2014; Kjellström et al., 2016).

Mutatis mutandis, there were observed increment in persons’ reliability measures to 0.64, 0.61, and 0.70 for KAP, respectively (**Table 4**) compared to those in the draft scale (**Table 2**). The separation index values were also in the range of > 1, but < 2, which were indications of the levels of consistency in the respondent behavior, hence, it was an indication of the instruments’ capability of differentiating between high and low-performance abilities of respondents (Kook and Varni, 2008; Bond and Fox, 2015). As a measure of consistency in behaviors, it implied that lapses made by the respondents were likely to affect the separation index value negatively, meaning that increasing sample size further might lead to a possible decrease in the index value (Bond and Fox, 2015). Nevertheless, the results of the present study showed that the low values of the index might partly be attributed to the item content, as the number of items in some of the constructs was few, and it has been reported that there is tendency of getting more reliable information on a construct conveying more facts when many relevant questions on such constructs are asked compare to just asking fewer questions (Chang et al., 2010; Golino et al., 2014).

The overall result of items fit index values for MNSQ and Zstd in the 18 items-scale (**Table 5**) after deletion of misfit items were all within the acceptable range of 0.6 to 1.5, and ± 2 respectively (Bond and Fox, 2015); and PTMEA Corr were all positive with acceptable items correlation strength to the constructs of the model.

Contrary to conventional method of analysis for instrument validation using CTT (Habing, 2003; Hogarty et al., 2005; DeVon et al., 2007; Garson, 2008; Golino et al., 2014), the outcome of the present study indicated an exploratory psychometric properties of the scale based on Rasch measurement model considering the recommended sample size within the least suggested range of between 64 and 144 respondents suggested by Linacre (1994) and Chen et al. (2014) for achieving 95% confidence that the item calibrations were within ± 0.5 logits. In order to obtain a robust item parameter estimates, and based on the reported positive relationship between incremental increase in sample size and precision of item fitness to Rasch model (Wasserman and Bracken, 2003; Smith et al., 2008; He and Wheadon, 2012; Bond and Fox, 2015), further calibration of the scale in a larger sample would be likewise conducted in future study.

## Study Limitations

The study which conducted using adequate sample population of healthcare workers working in PHC facilities was to establish exploratory assessment of the instrument’s validity and reliability using respective targeted study population and not to extrapolate the results as evidence in larger populations. For substantiating the results, a supplementary study would be carried out strictly adhering to sample size requirement for employing Rasch modeling. Additionally, as this instrument was designed mainly for trained PHC workers involved in uncomplicated malaria treatment, therefore, it would be of little importance in assessing respondents’ management practices for severe malaria, and also among those working in secondary and tertiary healthcare facilities. Since, the study was conducted only in Plateau state, Nigeria, to enable its generalization in the country and beyond, there would be a need for conducting the same study across different healthcare facilities across the country and beyond.

## Conclusion

This study has shown the usefulness of Rasch measurement model in assessing the validity and reliability of HKAPIUM scale. The goodness of fit indices indicated that the constructs components of the instrument satisfied the Rasch measurement model requirement, and was considered having acceptable reliability and validity as a measurement scale for PHC workers’ KAP in the management of uncomplicated malaria.

## Ethics Statement

This study was carried out in accordance with the recommendations of Nigeria and World Health Organisation guidelines, with permission granted by the Joint Research Review and Ethics Committee (REC), Research Management Centre (RMC), MAHSA University, Malaysia (Ref. number: RMC/EC01/2016; Dated 25/11/2016) with written informed consent from all subjects. All subjects gave written informed consent in accordance with the Declaration of Helsinki. The protocol was approved by the REC, RMC, MAHSA University.

## Author Contributions

All authors actively participated in the study. NJ **c**onceived and designed the study, collected the data, analyzed and wrote the first draft of the manuscript. MD participated in the design of the draft study instrument and data collection. SA and NJ managed the data analysis aspect of the study. NI critically reviewed the manuscript content. The final write-up was read and approved by all the authors.

## Conflict of Interest

The authors declare that the research was conducted in the absence of any commercial or financial relationships that could be construed as a potential conflict of interest.
